# Combination Treatment of Periungual Warts

**DOI:** 10.4103/0974-2077.41154

**Published:** 2008-01

**Authors:** Sukhdeo Patidar

**Affiliations:** *Baroda Skin Clinic, Saraswati Complex, Nanjalpur, Vadodara, Gujarat, India*

**Keywords:** Verruca, electrodessication, trichloroacetic acid

## Abstract

Periungual wart presents challenge for management in view of their frequent recurrence. The case report demonstrates the efficacy of a combination treatment of electrodessication and trichloroacetic acid application.

## INTRODUCTION

Warts or verruca vulgaris[1] caused by *human papilloma virus* are of many variants depending on location on the skin. Periungual warts are the warts around the nails, which are common in nail biters. In the beginning, these are pinhead in sized, shiny, smooth, translucent and usually discrete. They grow in weeks or months to pea size, become rough, dirty brown grey or black and horny. These warts become fissured, inflammed and tender. They may recur after treatment.

## CASE REPORT

**Figure 1:**
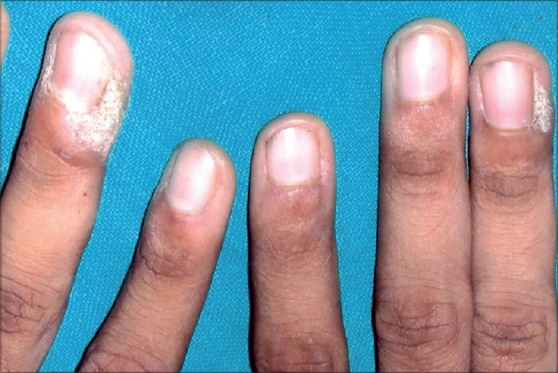
Periungual warts before treatment

A 24-year-old male had periungual wart of 2 years duration over right ring finger and left middle finger nails [Figure 1]. He was a habitual nail biter. Local anaesthesia was achieved by ring block and local infiltration. A torniquet was applied to minimise blood loss. Electrodesiccation was done as per the standard procedure[2] and lesion was curetted. Firm pressure was applied for 5 min. Once the bleeding was controlled, trichloroacetic acid (TCA), 30% solution was applied.

**Figure 2:**
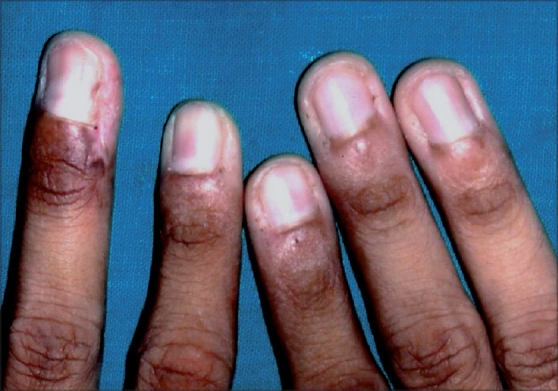
Follow-up after 1 year reveals no recurrence

Pressure dressing was applied, which was changed on second and seventh day. Systemic antibiotics and analgesics were prescribed for 7 days. Nail biting was strongly discouraged. After removal of dressing patient was advised to apply povidone-iodine ointment for 5 days. One year follow-up shows no recurrence [Figure 2].

## DISCUSSION

Prognosis of warts is variable due to unpredictable response to various therapeutic modalities namely, electrodesiccation, cryotherapy, chemical cauterization with salicylic acid, lactic acid, TCA, podophyllin and laser. The choice of treatment modality depends on location, size, number, secondary infection, pain and tenderness, age, sex and previous treatment. Recurrences are common with all the modalities. In the case reported here, combination of two methods, i.e., electrodesiccation and chemical cautery with TCA was used. This has given satisfactory results with no recurrence.
